# Local-scale structures across the morphotropic phase boundary in PbZr_1−*x*_Ti*_x_*O_3_

**DOI:** 10.1107/S2052252517016633

**Published:** 2018-01-01

**Authors:** Nan Zhang, Hiroko Yokota, A. M. Glazer, D. A. Keen, Semën Gorfman, P. A. Thomas, Wei Ren, Zuo-Guang Ye

**Affiliations:** a Electronic Materials Research Laboratory, Key Laboratory of the Ministry of Education and International Center for Dielectric Research, Xi’an Jiaotong University, Xi’an 710049, People’s Republic of China; b Department of Chemistry and 4D LABS, Simon Fraser University, 8888 University Drive, Burnaby, British Columbia V5A 1S6, Canada; c Department of Physics, Chiba University, 1-33 Yayoi-cho, Inage-ku, Chiba City 263-8522, Japan; d Department of Physics, University of Oxford, Parks Road, Oxford OX1 3PU, England; e Department of Physics, University of Warwick, Gibbet Hill Road, Coventry CV4 7AL, England; f ISIS Facility, Rutherford Appleton Laboratory, Harwell Campus, Didcot OX11 0QX, England; g Department of Materials Science and Engineering, Faculty of Engineering, Tel Aviv University, Tel Aviv 69978, Israel

**Keywords:** PZT, morphotropic phase boundary, local structure, polarization rotation

## Abstract

Lead zirconate titanate (PZT) is one of the most widely studied piezoelectric materials, mainly because of its ‘mysterious’ relationship between the so-called morphotropic phase boundary (MPB) and its strong piezoelectric coupling factor. Using results from a pair distribution function analysis, this paper examines how the complex local structure in PZT affects the long-range average structure across the MPB. A monoclinic M_*C*_ type structure is discovered in PZT. A first-order transformation between the monoclinic M_*A*_ and M_*C*_ components in both the average and local structures explains the sudden change in piezoelectric effect around these compositions. The role of polarization rotation in the enhancement of the piezoelectric properties is discussed with respect to the composition of PZT. The structure–property relationship that is revealed by this study explains the unique properties of PZT, and may be applicable in the design of new MPB-type functional materials.

## Abstract 2

Using neutron total scattering and pair distribution function analysis, this work discusses how the complex local structure in lead zirconate titanate affects the long-range average structure across the morphotropic phase boundary, and its influence on the unique piezoelectric properties.

## 1. Introduction   

The global commercial market for piezoelectric ceramics is several billions of dollars per annum (Innovative Research and Products Inc., 2013 ▸), among which the perovskite lead zirconate titanate solid solution (PbZr_1−*x*_Ti*_x_*O_3_, PZT) is the most widely used. Perovskite materials are well known for their structural flexibility: changes in temperature, pressure, electric field, stress *etc.* induce distortions from the parent cubic phase. Although these structural distortions are usually very small, they are the origin of various physical properties that are used in everyday life, as well as in industrial, military and high-end medical applications.

The piezoelectricity of PZT is particularly connected with its unique crystal structure, because of the presence of the so-called morphotropic phase boundary (MPB) (Jaffe *et al.*, 1954 ▸). The MPB (0.48 ≤ *x* ≤ 0.50 at room temperature) separates a Zr-rich rhombohedral (R) phase and a Ti-rich tetragonal (T) phase. The existence of a bridging monoclinic M_*A*_ structure at the MPB is now well accepted (Noheda *et al.*, 1999 ▸). According to Vanderbilt & Cohen (2001 ▸), the M_*A*_ and M_*B*_ structures (space group *Cm*) have cation displacements that lie on the {110} mirror planes, between the 〈111〉 and 〈001〉 directions or the 〈111〉 and 〈110〉 directions, respectively. The M_*C*_ (space group *Pm*) cation displacements lie on the {100} mirror planes. This latter structure type has so far not been discovered or discussed in PZT, but it was found in PMN-PT (Zekria & Glazer, 2004 ▸; Noheda *et al.*, 2002 ▸). Note that any change between M_*A*_ (or M_*B*_) and M_*C*_ structures must be of first order, as the displacements lie on different mirror planes and the corresponding space groups *Cm* and *Pm* are not group–subgroup related.

The existence of the *Cm* structure makes it possible for the polarization to rotate continuously from 〈110〉 to 〈001〉. This is consistent with the suggestion that the large piezoelectricity at the MPB arises from the rotation of the polarization vector under applied mechanical stress or electric field (Damjanovic, 2005 ▸; Fu & Cohen, 2000 ▸), which is known as the main contributor to the intrinsic effect. It should be noted that the boundary between monoclinic and rhombohedral regions is not observed at long length scales (Zhang *et al.*, 2014 ▸). Another possible contributor to the overall piezoelectric property is the extrinsic effect, which is the piezoelectric response arising from domain wall motion and domain volume exchange under electrical or mechanical load (Jones, 2007 ▸).

Nowadays, the discovery of new environmentally friendly lead-free piezoelectrics mostly relies on finding materials with an MPB similar to that in PZT. However, neither the MPB structure at the atomic scale nor the phase transformations across it are yet ‘crystal clear’. Various structural studies reveal that PZT has a more complex structure than many other perovskites, involving phase coexistence (Cox *et al.*, 2005 ▸; Frantti, 2008 ▸; Yokota *et al.*, 2009 ▸; Zhang *et al.*, 2011 ▸; Gorfman *et al.*, 2011 ▸) and complicated domain structures (Jones *et al.*, 2006 ▸; Theissmann *et al.*, 2007 ▸). This complexity is usually determined indirectly from conventional diffraction methods, revealing the long-range (average) structure only. The limited ability of average structure determination raises several questions. For instance, the low-symmetry diffraction pattern may be explained by a specific domain structure arrangement, described by the so-called adaptive phase model (Wang, 2007 ▸). In this model, the monoclinic phase does not exist as a real phase, but as the result of overlapping rhombohedral or tetragonal nanodomains, which makes it essentially an extrinsic contribution only. Therefore, it is important to extend the structural study to the local scale. In this case, the short correlation length, which extends over only a few unit cells, is smaller than the size of even one nanodomain. Since the local and long-range structures may differ, a full understanding of PZT must be based on the combined study of both, especially in the MPB region.

Information about short-range order can be obtained either from a study of diffuse scattering, usually from single crystals, or by total scattering from polycrystalline samples including both the diffuse and Bragg intensities (Keen & Goodwin, 2015 ▸). The Fourier transform of the total scattering gives the pair distribution function (PDF), from which one derives the weighted sum of the normalized probabilities of finding two atoms a certain distance apart (Keen, 2001 ▸). Although local symmetry-lowering and order–disorder phase transitions were discovered in perovskites as early as the 1970s (Comès *et al.*, 1970 ▸), it is only recently – thanks to the rapid development of instrumentation and software for total scattering – that it has become possible to study the complex local structures in various lead-based piezoelectrics and the relationships with their properties. A few PDF (Teslic *et al.*, 1996 ▸; Egami *et al.*, 1998 ▸; Zhang *et al.*, 2014 ▸) and diffuse scattering (Burkovsky *et al.*, 2012 ▸) studies of PZT have revealed local clusters with complex structures, mostly in the rhombohedral phase or near the MPB region. For example, our recent study (Zhang *et al.*, 2014 ▸) reported a short-range monoclinic M_*B*_–M_*A*_ structural change as a function of Zr concentration in the rhombohedral region. This suggested that the rotation of the Pb displacement vector from [110] towards the [001] pseudocubic crystallographic directions on going from M_*B*_ to M_*A*_ structures plays an important role in the increase in piezoactivity on the Zr-rich side of the MPB. Bogdanov *et al.* (2016 ▸) performed density functional theory (DFT) calculations of the local structure of various compositions of PZT. They especially searched for the structural models of PZT (*x* = 0.4) and compared them with our PDF data. Local Pb displacements were found in almost all monoclinic (M_*A*/*B*_ and M_*C*_) mirror planes, and they proposed that the variation in Pb displacement has a strong correlation with the Ti/Zr environment. While much attention has been paid to the study of the rhombohedral and MPB compositions, the local structure on the tetragonal side of the MPB is still largely unexplored.

The structure of Ti-rich PZT is traditionally considered to be tetragonal (space group *P*4*mm*), similar to PbTiO_3_. Unlike the ‘missing boundary’ between R and M regions, the M and T phase boundary is always observable in ceramics and single crystals (Noheda *et al.*, 2000 ▸). However, some indirect observations reveal that the local structure of the tetragonal phase may also be complex. Rossetti *et al.* (1999 ▸) observed asymmetric Bragg reflections in X-ray diffraction and explained this in terms of microstrain line broadening. High-resolution neutron diffraction shows a similar result for PZT with *x* = 0.6 (Zhang *et al.*, 2011 ▸). However, the incorporation of anisotropic microstrain line broadening in Rietveld refinement still could not fit the peak shape well. Frantti *et al.* (2013 ▸) reported unusual splitting in the *A*_1_(TO) and the four *E*(TO) Raman modes in a Pb(Zr_0.35_Ti_0.65_)O_3_ single crystal. They suggested that the short-range order is different from that seen in the average *P*4*mm* phase. They introduced a statistical model of Pb displacements along the 〈11*z*〉 directions (note that all crystallographic directions in this paper are with respect to the pseudocubic perovskite axes), which agrees with the short-range order model of Glazer *et al.* (2004 ▸). Polarized-light microscopy studies of the tetragonal phase in single crystals show that the crystals appear to be optically isotropic. This has been explained by the overlapping of tetragonal nanodomains along the 〈100〉 directions (Bokov *et al.*, 2010 ▸).

Here, we present a study of the local structural changes from the rhombohedral into the tetragonal regions of the PZT phase diagram across the MPB. The PDF data were obtained by neutron total scattering measurements and analysed by the reverse Monte Carlo (RMC) method (McGreevy, 2001 ▸). Most importantly, we show that short-range monoclinic M_*A*_ and M_*C*_ cation displacements exist in the tetragonal compositions. This result complements the previous knowledge about rhombohedral PZT, where short-range M_*B*_/M_*A*_ cation displacements exist. It also gives a new perspective on the R–M–T phase transition that is different from the traditional view.

## 2. Experimental   

The ceramic samples were prepared by the conventional mixed-oxide method. Details of the sample preparation conditions can be found in the work by Yokota *et al.* (2009 ▸).

The total neutron scattering experiments were carried out at ISIS (Rutherford Appleton Laboratory) on the GEM (general materials) diffractometer. The details of data correction, RMC modelling, atom position calculation and stereographic projections can be found in the work by Zhang *et al.* (2014 ▸). To improve the statistics of the results, the data for each composition were RMC modelled 100 times.

During the RMC process, starting models with different box shapes and different initial atom positions were both tried. Because of the randomization process of the Monte Carlo method, the initial positions do not affect the final results. For example, structural models with Pb atoms that are displaced in different crystallographic directions [*e.g.* on the (110) plane or the (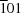) plane], or without any displacements, provide similar results regarding the local symmetry. However, different box shapes may significantly affect the final structure. A large distortion from a pseudocubic structure may lead to biased results. Therefore, in this study, the starting models were chosen to be as close to cubic as possible, whilst still being able to refine the Bragg data at the same time.

## 3. Results and discussion   

### 3.1. Local structure   

**Figure 1:**
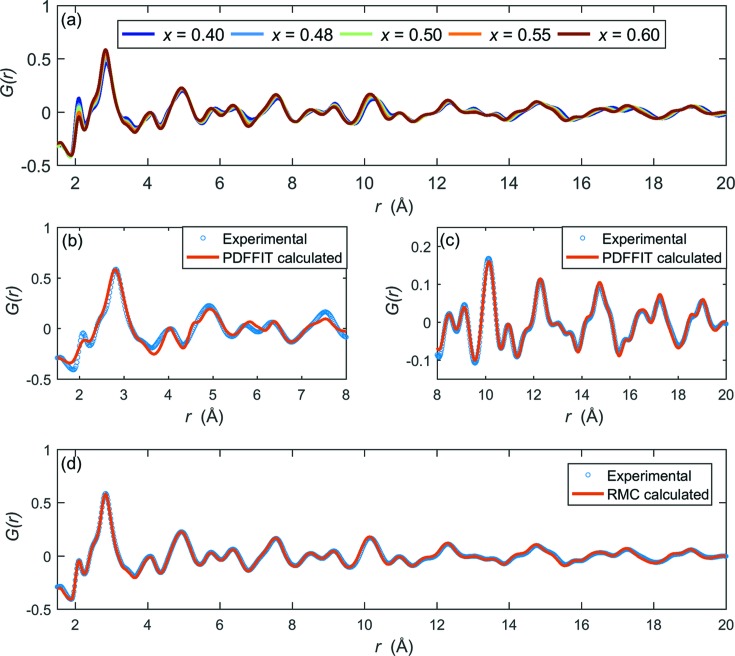
(*a*) PDF data for PZT *x* = 0.40–0.60. (*b*), (*c*) Small-box modelling of the PZT *x* = 0.60 (tetragonal) PDF data using *PDFFIT*. The fitting range is restricted to 1.5–8 Å as very short-range and 8–20 Å, respectively. (*d*) Big-box modelling of the PZT60 (tetragonal) PDF data using the RMC method.

We chose four powder samples (*x* = 0.48, 0.50, 0.55 and 0.60) for the total scattering experiments. The overall radial distribution functions, *G*(*r*), are presented in Fig. 1 ▸(*a*). We also include one rhombohedral composition (*x* = 0.40; Zhang *et al.*, 2014 ▸) in the figure for comparison. As in the rhombohedral compositions, a positive Zr—O peak occurs at 2.1 Å and a negative Ti—O peak (arising from the fact that the neutron scattering length for Ti is negative) is found at 1.86 Å. This differentiates between Zr and Ti cations, and cannot be refined in normal diffraction studies using Bragg reflections alone. Increasing the Ti concentration leads to a continuous intensity change for different peaks in the *G*(*r*) plot. At the same time there is no sign of any abrupt phase change. The *G*(*r*) patterns are first refined with a small-box modelling method using the software *PDFFIT* (Egami & Billinge, 2012 ▸; Farrow *et al.*, 2007 ▸). Figs. 1 ▸(*b*) and 1 ▸(*c*) show typical examples of *PDFFIT* models using a single-phase tetragonal structure for PZT *x* = 0.6. The fitting procedure was carried out using different ranges, 1.5 to 8 Å and 8 to 20 Å, separately. Although a single tetragonal model can reproduce the experimental data well above 8 Å (presented in Fig. 1 ▸ *c*), there are several peaks which are not fitted in the very short range below 8 Å, as shown in Fig. 1 ▸(*b*). Since this composition is close to the MPB, a single monoclinic phase was also tried with *PDFFIT*, but it too did not explain the very short-range pattern in the PDF (see Fig. S1 in the supporting information). This indicates that there is a more complex local structure for PZT. The *PDFFIT* results of other compositions are similar to that for *x* = 0.60 (see Figs. S2–S4 in the supporting information). To explore this complexity, we changed to big-box modelling with the RMC method. Fig. 1 ▸(*d*) shows that the *G*(*r*) pattern with RMC refinements gives a good fit to the experimental data. The structure factor and Bragg profiles were refined at the same time (see Fig. S5 in the supporting information). The PDF fits for other compositions are shown in Fig. S6 in the supporting information.

**Figure 2:**
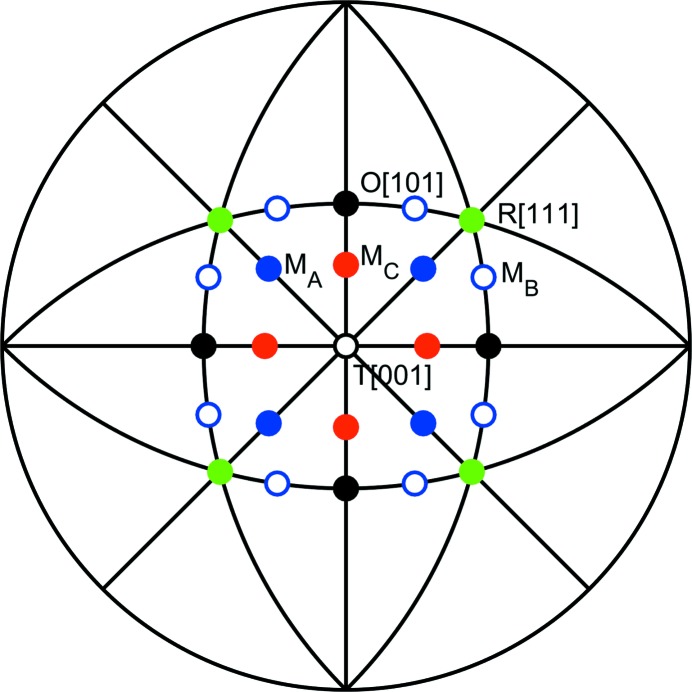
Stereographic projection of the major pseudocubic directions in perovskites, viewed down [001]. Directions closest to [001] are marked as dots. The green solid circles refer to the 〈111〉 directions (three-fold symmetry axes for rhombohedral cation displacements), black solid circles mark the 〈110〉 directions (two-fold axis of orthorhombic symmetry) and black open circles refer to the [001] directions (four-fold axis for tetragonal symmetry). Blue solid and open circles represent possible M_*A*_ and M_*B*_ directions, respectively, on the {110} monoclinic mirror planes. Red solid circles represent possible M_*C*_ directions on the {100} monoclinic mirror planes.

In order to have statistically significant estimates for the cation displacement directions at the unit-cell scale, we performed around 100 RMC refinements using 10 × 10 × 10 pseudocubic unit cells for each set of data. A bigger box was also tried, and the results were similar. The initial structures for all the RMC refinements were derived from Rietveld refinement against the Bragg profiles. These initial structures are monoclinic *Cm* for *x* = 0.48 and *x* = 0.50 data, and tetragonal *P*4*mm* for *x* = 0.55 and *x* = 0.60 data. After the RMC refinements, we extracted the atomic positions and plotted the distributions of the directions of atomic displacements (relative to a position defined by the geometric centres of the six oxygen atoms surrounding the *B* cations or the 12 oxygen atoms surrounding the *A* cations). A schematic stereographic projection viewed down the pseudocubic [001] direction with cation displacements towards directions corresponding to T, R, M_*A*_, M_*B*_ and M_*C*_ structures is shown in Fig. 2 ▸.

**Figure 3:**
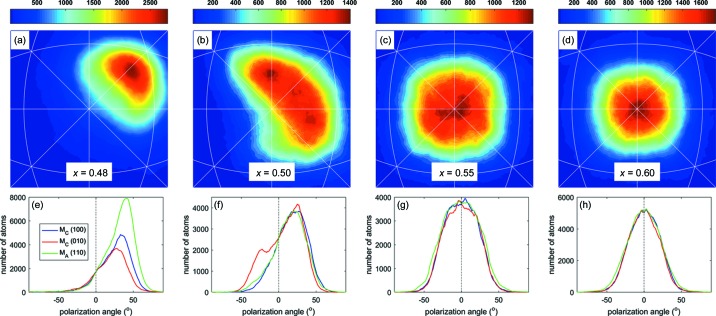
Histograms of the Pb atom displacements in PZT (*x* = 0.48, 0.50, 0.55 and 0.60), shown on stereographic projections in panels (*a*)–(*d*) and in the form of polarization rotation angles in panels (*e*)–(*h*). The colour scheme in the top figures reflects the number of Pb atoms in the supercell, displaced in given crystallographic directions. The bottom figures are one-dimensional distribution profiles of the Pb atom displacement angles on the specific mirror planes away from the [001] direction.

The top of Fig. 3 ▸ shows the distributions of local Pb displacement directions in the central area of the stereographic projection. At the bottom of Fig. 3 ▸ are the corresponding one-dimensional distributions of Pb displacement directions on the specific mirror planes. The measured angle is the displacement vector from the [001] direction. For *x* = 0.48 (Fig. 3 ▸ *a*), the Pb atoms are mostly in the M_*A*_ directions, in agreement with the symmetry of the average structure. The maximum intensity of the distribution is parallel to the [0.68 0.68 1] direction, 44(±1)° away from [001] (the error bar is calculated as the estimated standard deviation of the angle distribution maxima for 100 RMC runs). In the one-dimensional distribution plot for *x* = 0.48, the Pb displacements are within the range of ±50°. Therefore the percentage of atoms displaced in the M_*B*_ direction is almost 0. When *x* = 0.50, the Pb displacements move closer to the tetragonal direction, with the centre of the distribution being parallel to [0.34 0.34 1], 26(±1)° away from [001]. At the same time, some Pb atoms are displaced on the (010) and (100) planes, with the local maxima also around 26° from [001]. In fact, the *x* = 0.50 RMC refinement process yields equal probabilities for M_*A*_ and M_*C*_ type displacements for Pb atoms among the different RMC refinements. This result suggests a coexistence of local M_*A*_ and M_*C*_ components at this boundary of the MPB region. Note that all previous attempts to add a secondary monoclinic component to model the average structure at this composition assumed that the local monoclinic order is only M_*A*_ (Zhang *et al.*, 2011 ▸; Frantti *et al.*, 2013 ▸; Glazer *et al.*, 2004 ▸; Wei *et al.*, 2016 ▸).

When *x* increases to 0.55–0.60, PZT enters the average ‘tetragonal region’ in that the diffraction pattern can be refined as tetragonal, sometimes with a minor second phase (Zhang *et al.*, 2011 ▸). In Fig. 3 ▸(*c*) (*x* = 0.55) there are still a large number of Pb atoms displaced on the M_*A*_ and M_*C*_ mirror planes, in the range of 5–10(±1)° away from [001]. For *x* = 0.60 in Fig. 3 ▸(*d*), most of the displacement directions are already very close to 0(±0.8)° from [001], but the maximum distributions are seen better in the one-dimensional profile in Fig. 3 ▸(*h*) where, as shown by the narrower width, there are fewer Pb directions exactly along [001]. Local Pb displacement vectors are found in multiple monoclinic mirror planes at this composition, similar to what has been suggested by Bogdanov *et al.* (2016 ▸). From the MPB region to the ‘tetragonal’ side, the correlation length for the M_*A*_ components decreases to form a short-range M_*A*_ structure, and at the same time there are a certain number of cations displaced along the M_*C*_ direction, forming a short-range M_*C*_ structure. This leads us to propose a structural model where the local monoclinic M_*A*_ and M_*C*_ ordering averages to form a long-range tetragonal symmetry, as observed by the Bragg diffraction patterns. The existence of a first-order transformation between M_*A*_ and M_*C*_ local structures explains why the boundary on the right-hand side of the MPB is so sharp. Further increasing the Ti concentration moves the Pb displacements closer to the [001] direction. For compositions higher than *x* = 0.60, the solid solution enters a ‘pure’ long-range tetragonal region.

**Figure 4:**
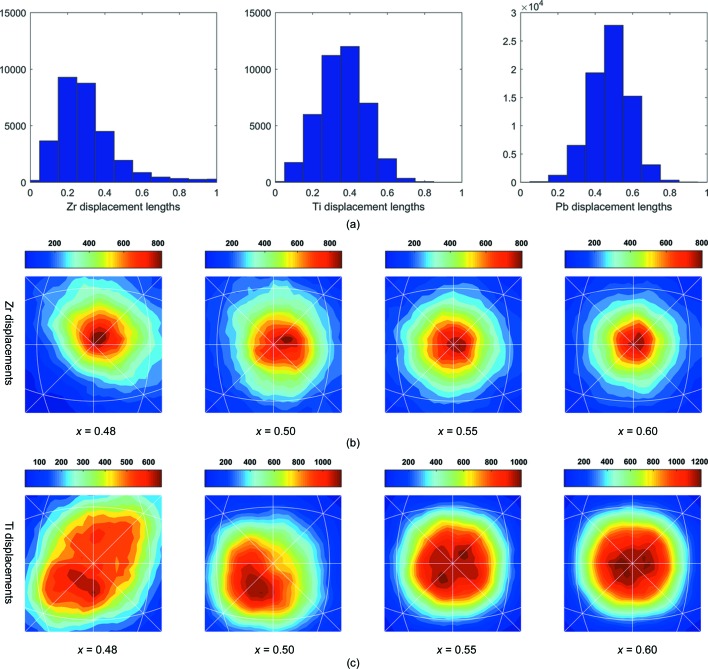
Quantitative analyses of *B* cation displacements. (*a*) Histograms of Zr and Ti displacement lengths for PZT (*x* = 0.50) as examples, and with an equivalent Pb histogram for comparison. (*b*), (*c*) Distributions of (*b*) Zr and (*c*) Ti atom displacement directions plotted on stereographic projections for different compositions across the MPB.

The *B*-cation displacements show quite different behaviour from the *A*-cation displacements. In addition, the Zr and Ti displacements are different from each other. For example, for *x* = 0.50, while most of the Pb atoms are displaced by ∼0.5 Å, Zr/Ti atoms are displaced by ∼0.2 and ∼0.4 Å, respectively (Fig. 4 ▸ *a*). Figs. 4 ▸(*b*) and 4 ▸(*c*) display the distributions of the displacement directions for Zr and Ti at different compositions. With a short displacement length and displacement directions distributed closely around [001], the Zr atomic displacements are more isotropic and the atom positions are very close to the cubic aristotype position. This has been reported in other local structure studies of PbZrO_3_ (Teslic & Egami, 1998 ▸) and in other compounds (Kuzmin *et al.*, 2000 ▸; Laulhé *et al.*, 2006 ▸; Rabuffetti & Brutchey, 2013 ▸). The tendency for Zr atoms to move less off-centre is consistent with what is seen in the antiferroelectric PbZrO_3_ and cubic BaZrO_3_ structures, both of which are centrosymmetric. In contrast with Zr, many Ti displacements are distributed in directions further away from [001] in the stereographic projection, indicating a higher level of displacive disorder. It is worth noting that there are a considerable number of Ti displacements accumulated on both local M_*A*_ and M_*C*_ mirror planes, similar to Pb. Our previous study of the Zr-rich side of the MPB (Zhang *et al.*, 2014 ▸) showed that local Ti-atom displacements are mostly accumulated around 〈111〉, consistent with average rhombohedral symmetry. Combining the two studies in the Zr-rich and Ti-rich compositions, the change from order to disorder in Ti happens when the monoclinic phase becomes dominant at *x* = 0.48. Similar displacive disorder has been found in BaTiO_3_-based materials (Senn *et al.*, 2016 ▸; Levin *et al.*, 2014 ▸), where the Ti atoms tend to be displaced along rhombohedral 〈111〉 directions within an average tetragonal symmetry. It is worth noting that, despite the higher displacive disorder level of Ti in comparison with Zr, this is not in conflict with the fact that Ti is a ‘ferro­electrically active’ *B* cation in perovskites. The local displacements have preferred directions, on the monoclinic mirror planes or towards the direction of the rhombohedral symmetry axis. These displacement directions with larger displacement lengths (than Zr) give switchable polarizations for Ti cations.

### 3.2. On the M_*A*_–M_*C*_ boundary   

Our RMC structural model for *x* = 0.48 clearly shows that most Pb, Zr and Ti atoms are displaced in the M_*A*_ directions and that these displacements vary in length. The local atomic displacements at the MPB composition seem to be the most ‘ordered’ in terms of local displacements, in agreement with the prediction made by Glazer *et al.* (2004 ▸). However, the powder diffraction pattern around this composition always gives complex profiles for Bragg reflections and has previously been refined using a mixture of two or three coexisting structures (Frantti, 2008 ▸; Zhang *et al.*, 2011 ▸). With our current findings on the local monoclinic structure, this can be explained by the coexistence of long-range monoclinic regions and long-range rhombohedral/tetragonal regions arising from local monoclinic distortions.

Moving away from the MPB on both sides, different types of lower-symmetry components are observed by PDF analysis. The length scale for the monoclinic order can be as small as a few unit cells. There have been reports of similar phenomena using convergent-beam electron diffraction (CBED), which covers a local region of the sample with sizes down to 5–10 nm. For example, Schierholz and co-workers performed CBED experiments on PZT ceramics with various compositions (Schierholz & Fuess, 2011 ▸; Schierholz *et al.*, 2008 ▸). Small probe sizes usually lead to the observation of lower symmetry in comparison with the average structure, as marked by the breakdown of the symmetry of a zone axis. In fact, in the tetragonal compositions of PZT ceramics, regions with {100} mirror planes and without a fourfold axis were frequently observed, which can be explained by the M_*C*_ structure. Local symmetry lowering was also found in other materials, such as PMN-PT (Kim *et al.*, 2013 ▸). These results agree very well with our local structural model.

**Figure 5:**
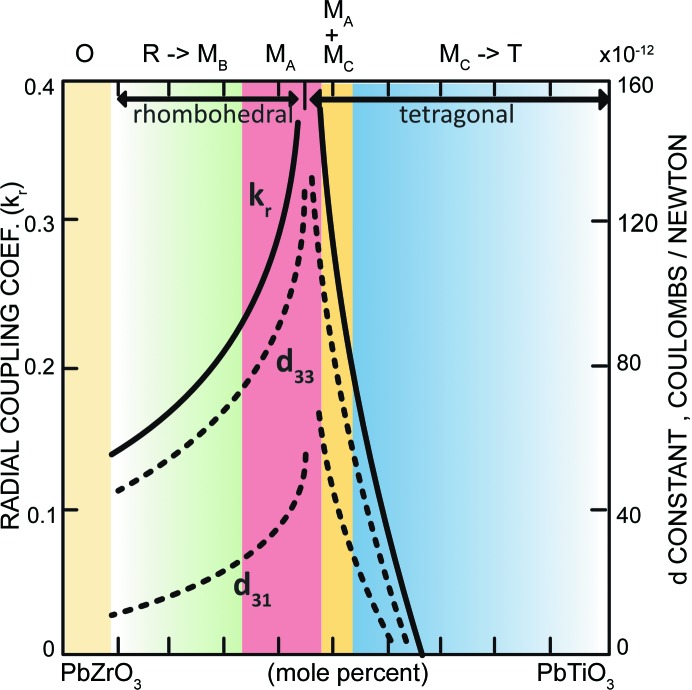
A summary drawing indicating the local structures of PZT at room temperature, together with a plot of the piezoelectric properties as a function of composition. [Redrawn from Jaffe *et al.* (1954 ▸). Copyright 1954, AIP Publishing LLC].

If one looks back into the history of the determination of the structures and their contributions to the piezoelectricity of PZT, there has always been a question over the crystal structures on either side of the MPB, which are not group–subgroup symmetry-related. The variation of the piezoelectric activity with composition, according to Jaffe *et al.* (1954 ▸), showed that maximum piezoactivity occurs at the MPB, coinciding with the R–T phase transition. In the bridging monoclinic phase model (Noheda *et al.*, 1999 ▸), the *Cm* space group is a subgroup of both *R*3*m* and *P*4*mm*. Therefore, the R–M and M–T phase transitions can both be continuous with monoclinic cation displacements moving from [111] to [001]. However, a more careful look at Jaffe’s piezoelectric measurements (Fig. 5 ▸) reveals rather different trends on the Zr-rich and Ti-rich sides of the MPB: the piezoelectricity rises smoothly on the Zr-rich side of the MPB but drops very abruptly on the Ti-rich side. We have already explained the trend on the Zr-rich side by the local M_*B*_–M_*A*_ change (Zhang *et al.*, 2014 ▸), where the Pb displacement direction rotates continuously on approaching the MPB. On the other hand, the M_*A*_ (*Cm*) and M_*C*_ (*Pm*) structures are not group–subgroup related, and so the structural change involves the cation displacements jumping from one mirror plane to another, despite the fact that they are all close to [001]. This conclusion seems to bring us back to the pre-bridging-phase era, where we have two non-group–subgroup related phases along a phase boundary. However, our model is obviously different in terms of explaining the properties: there are significant numbers of local monoclinic components on both sides of the MPB.

### 3.3. Intrinsic and extrinsic contributions to piezo­electricity   

On either side of the MPB, the directions of Pb displacements in adjacent monoclinic unit cells within each individual crystallite are uncorrelated and they average out to give an overall rhombohedral (Zr-rich side) or tetragonal (Ti-rich side) structure. As the Zr/Ti ratio approaches the MPB, these Pb displacements begin to correlate, creating islands of longer-range monoclinic structure within the dominant matrix of the average structure. The volume of these long-range ordered monoclinic regions maximizes at the MPB to become large enough to diffract as a distinct monoclinic phase. The growth of these long-range monoclinic regions results in an increase in the number of domain walls separating the 24 types of possible domain. Also, because all monoclinic unit cells are close to being metrically cubic in perovskites, the domains may occur frequently. The extrinsic contribution to the piezoelectricity is related to the domain wall density and mobility, and so the formation of a monoclinic domain pattern potentially leads to an increase in extrinsic contributions. Overall, as the number of domains increases and at the same time the domains become more fine-grained, the extrinsic component is likely to dominate the piezoelectricity, especially on approaching the MPB.

Correspondingly, the local M_*B*_–M_*A*_ change on the Zr-rich side and the local M_*A*_–M_*C*_ coexistence on the Ti-rich side give freedom for polarization rotation of the Pb vector, which increases its intrinsic contribution to the piezoelectricity in addition to the extrinsic component. However, the rotation angles on either side of the MPB are different. From the PDF results, on the Zr-rich side of the MPB, over a large composition range (from *x* ≃ 0.33 to *x* = 0.50), the polarization (derived from Pb displacements) rotates by almost 55° in the M_*A*_ component. As *x* increases, this smoothly develops into the MPB region. Correspondingly in the M_*C*_ component, the polarization rotation disappears very quickly over a small change in composition (*x* = 0.50 to *x* < 0.60) beyond the MPB.

**Figure 6:**
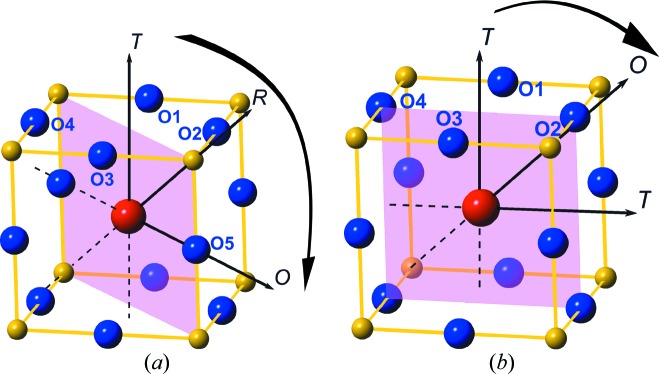
Schematic diagrams of the polarization rotation paths for Pb displacement vectors in the monoclinic mirror planes in the (*a*) M_*A*_/M_*B*_ and (*b*) M_*C*_ structures. The large red atom at the centre of the unit cell is Pb, while the blue atoms are the surrounding O atoms of the PbO_12_ cage. The small yellow atoms are the *B*-site cations Zr or Ti. Arrows indicate possible vector directions.

Such different behaviour of the cation displacement vectors in the different types of monoclinic structure has also been discussed based on theoretical calculations (Damjanovic, 2005 ▸; Fu & Cohen, 2000 ▸). The origin of these differences can be explained qualitatively as follows. Fig. 6 ▸(*a*) shows the journey that the Pb vector makes on the Zr-rich side on rotating from the T direction towards the O direction *via* the R direction as the Zr content increases. When along the T direction, the Pb atom points into a large space surrounded by four oxygen atoms (O1, O2, O3 and O4 in the figure), and is therefore relatively free to move on application of an external stimulus (stress, electric field *etc*). On increasing the Zr content it is free to rotate on the {110} mirror plane, passing through a large angle between O2 and O3 before pointing directly towards O5 at high Zr content. It is well known that Pb—O bonding is strong because of the 6*s* ^2^ lone pair in the Pb cation, and so this represents a gradual change from an ionic state towards a more covalent state. As the Pb displacement vector rotates towards O5, the increase in covalent strength blocks further rotation. At the same time, our refinements show that the magnitude of the Pb displacement hardly changes with composition on the Zr-rich side. Taken together, this explains why the polarization rotation changes only gradually when in the M_*B*_ structure but then increases rapidly after transforming to the M_*A*_ structure. Fig. 6 ▸(*b*) shows a similar journey for the Pb vector in the M_*C*_ structure. At the MPB, the Pb displacement vector is close to one of the T directions, but on further rotation, as the Ti concentration increases away from the MPB, it starts to experience interaction with atom O2 along the orthorhombic direction. Again this makes the Pb—O bond more covalent and stops further rotation of the Pb displacement vector, which suppresses the intrinsic contribution from polarization rotation to the piezoelectric effect. As a result, the interaction with atom O2 occurs only after a few degrees of rotation with increasing Ti concentration. In a sense, therefore, the M_*C*_ journey is similar to the M_*B*_ case on the Zr-rich side, with the M_*A*_ structure being the most important intrinsic contributor.

## 4. Conclusions   

From the above discussions, it is evident that different proportions of intrinsic and extrinsic contributions to the overall piezoelectric effect exist on either side of the MPB, although we cannot determine the precise amounts from PDF analyses. The extrinsic effect rises to a maximum at the MPB, where many fine-scale monoclinic domains are expected. At the same time, the intrinsic component is superimposed, growing on the Zr-rich side towards the MPB and falling rapidly on the Ti-rich side. The combination of the two effects explains well the asymmetry with composition observed in the piezoelectric response shown in Fig. 5 ▸. This further reveals the close relationship between short-range and long-range monoclinic structures and enhancement of the piezoelectric properties of PZT at the MPB.

## Supplementary Material

Additional figures. DOI: 10.1107/S2052252517016633/ti5011sup1.pdf
