# Supplementary material for: Local-scale structures across the morphotropic phase boundary in PbZr1−xTixO3
Source: IUCrJ. 2018 Jan 1;5(Pt 1):73–81. doi: 10.1107/S2052252517016633 (PMC5755579; doi:10.1107/S2052252517016633)
Supplement: Supplementary file 1 [file m-05-00073-sup1.pdf]

IUCrJ

Volume 5 (2018)

Supporting information for article:

Local-scale structures across the morphotropic phase boundary in $PbZr_{1-x}Ti_{x}O_3$

Nan Zhang, Hiroko Yokota, A. M. Glazer, D. A. Keen, Semën Gorfman, P. A. Thomas, Wei Ren and Zuo-Guang Ye

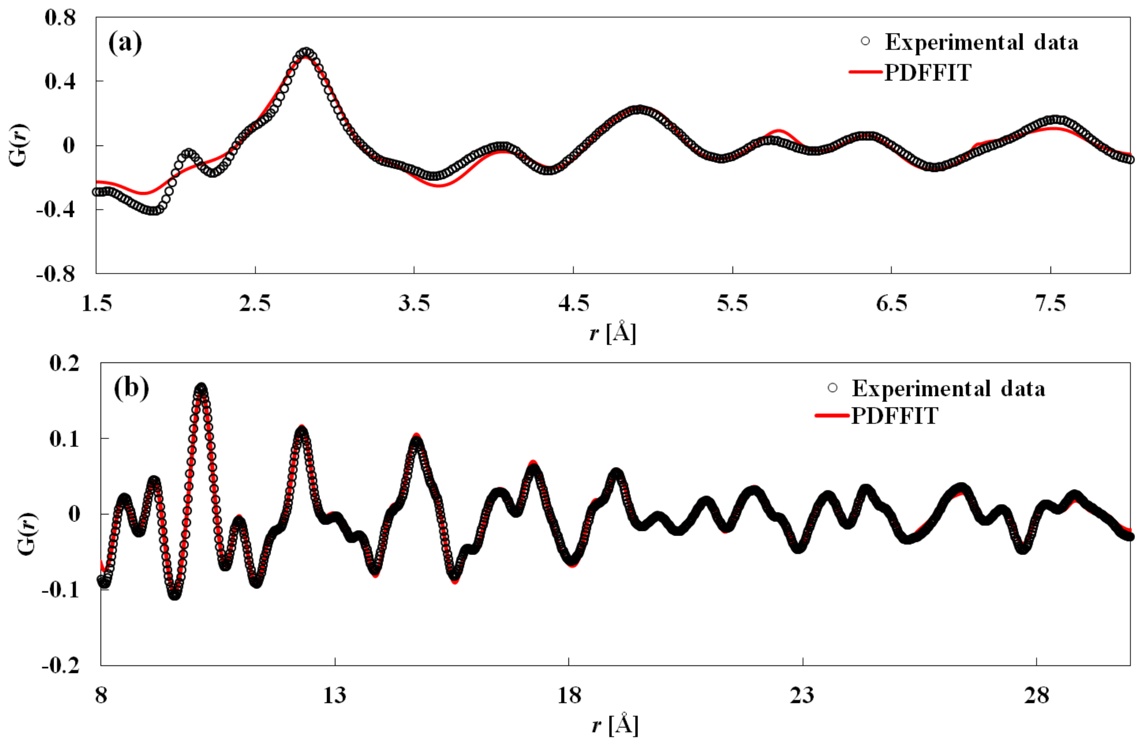

Fig. S1 *PDFFIT* for PZR, $x = 0.60$.

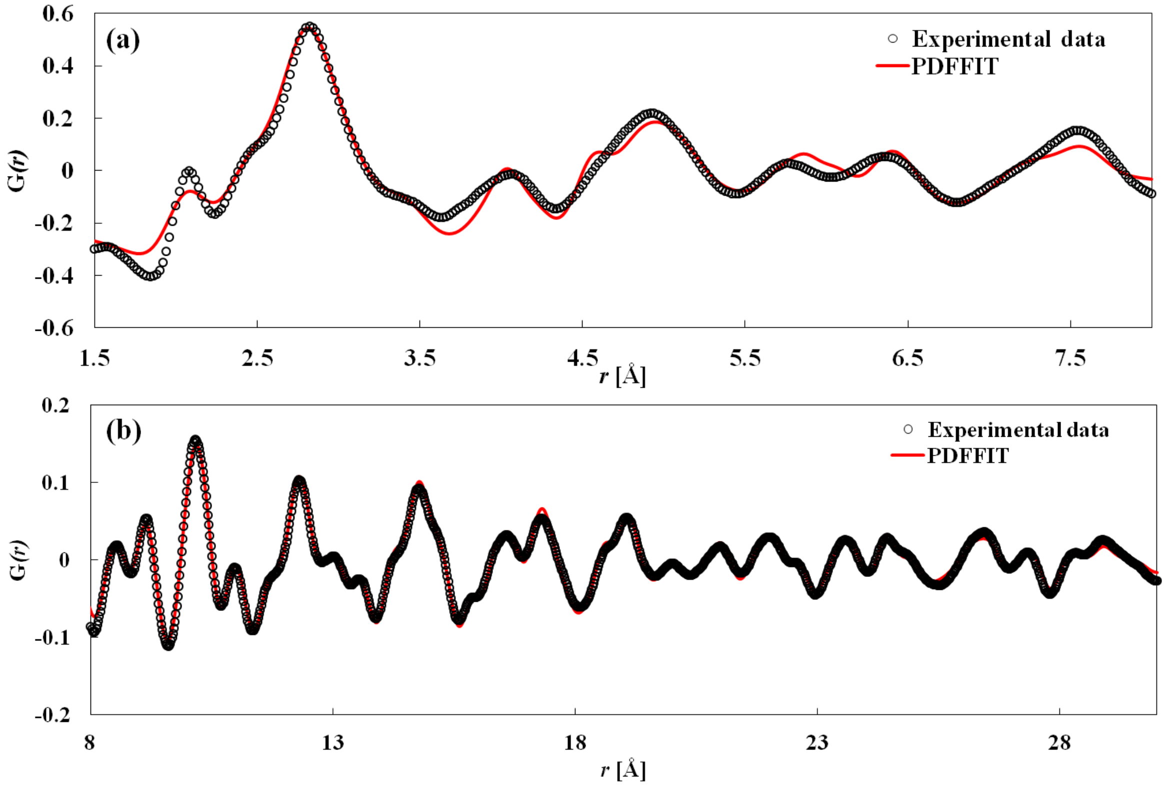

Fig. S2 *PDFFIT* for $x = 0.55$.

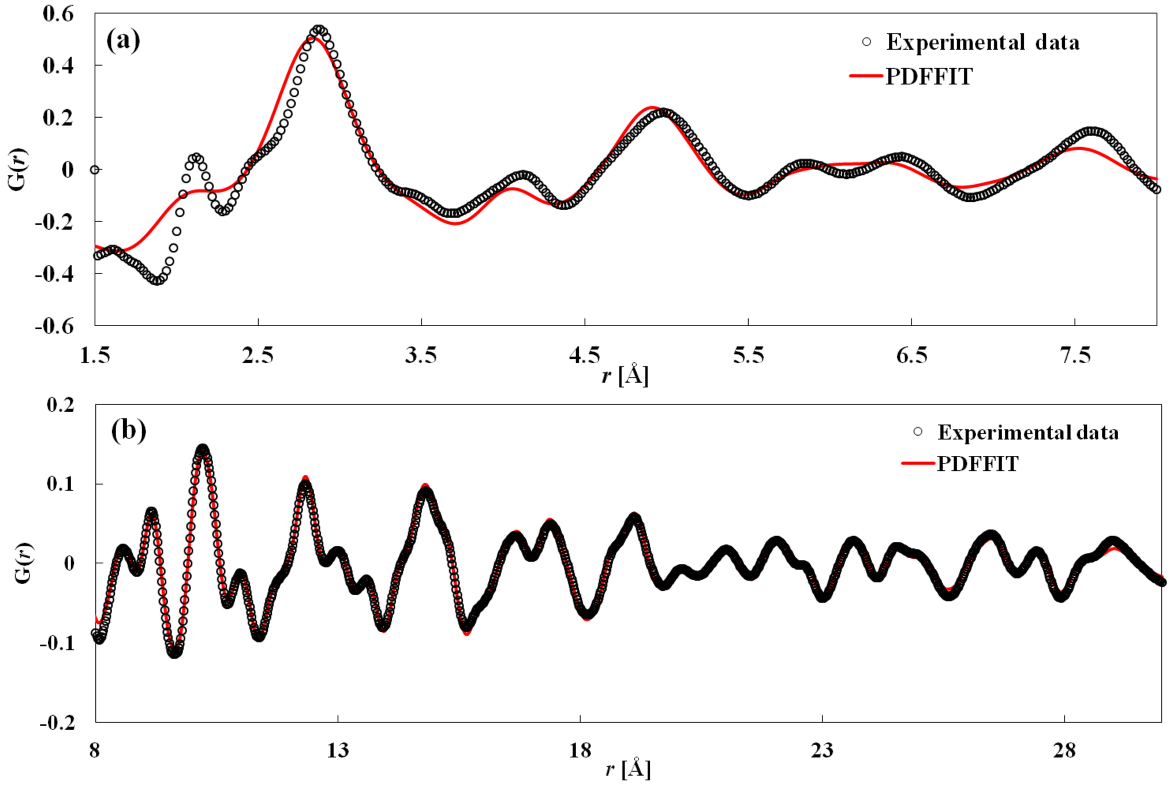

Fig. S3 *PDFFIT* for x = 0.50.

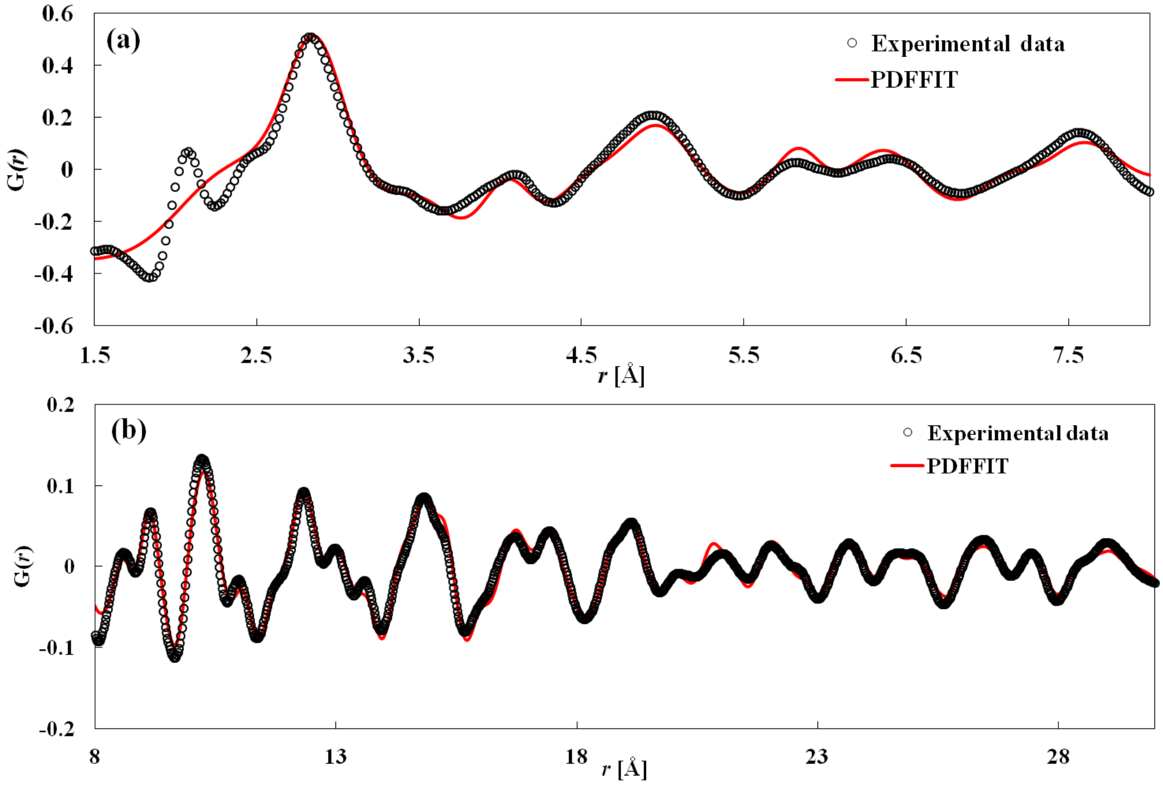

Fig. S4 *PDFFIT* for x = 0.48.

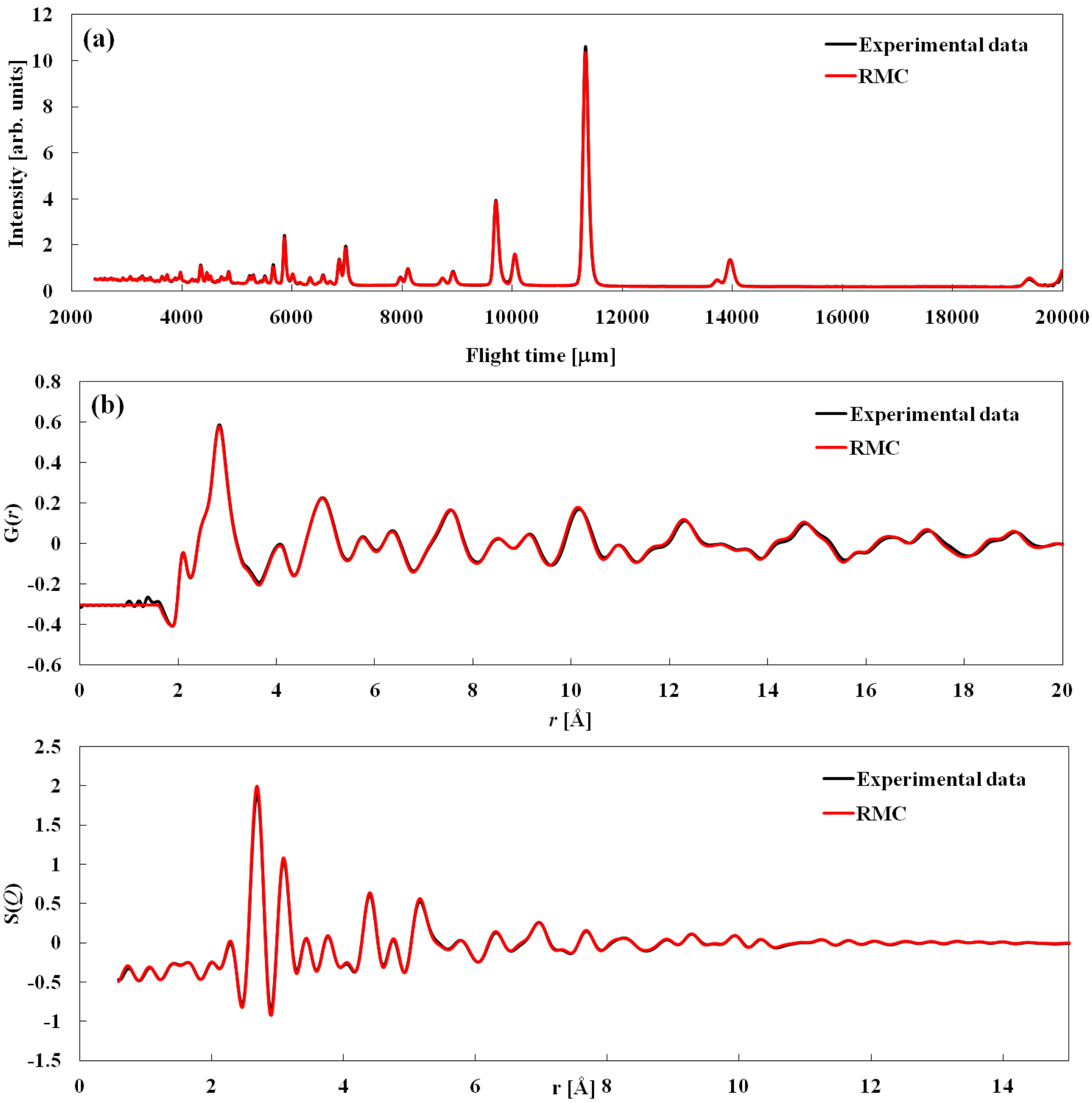

Fig. S5 RMC results for $x = 0.60$.

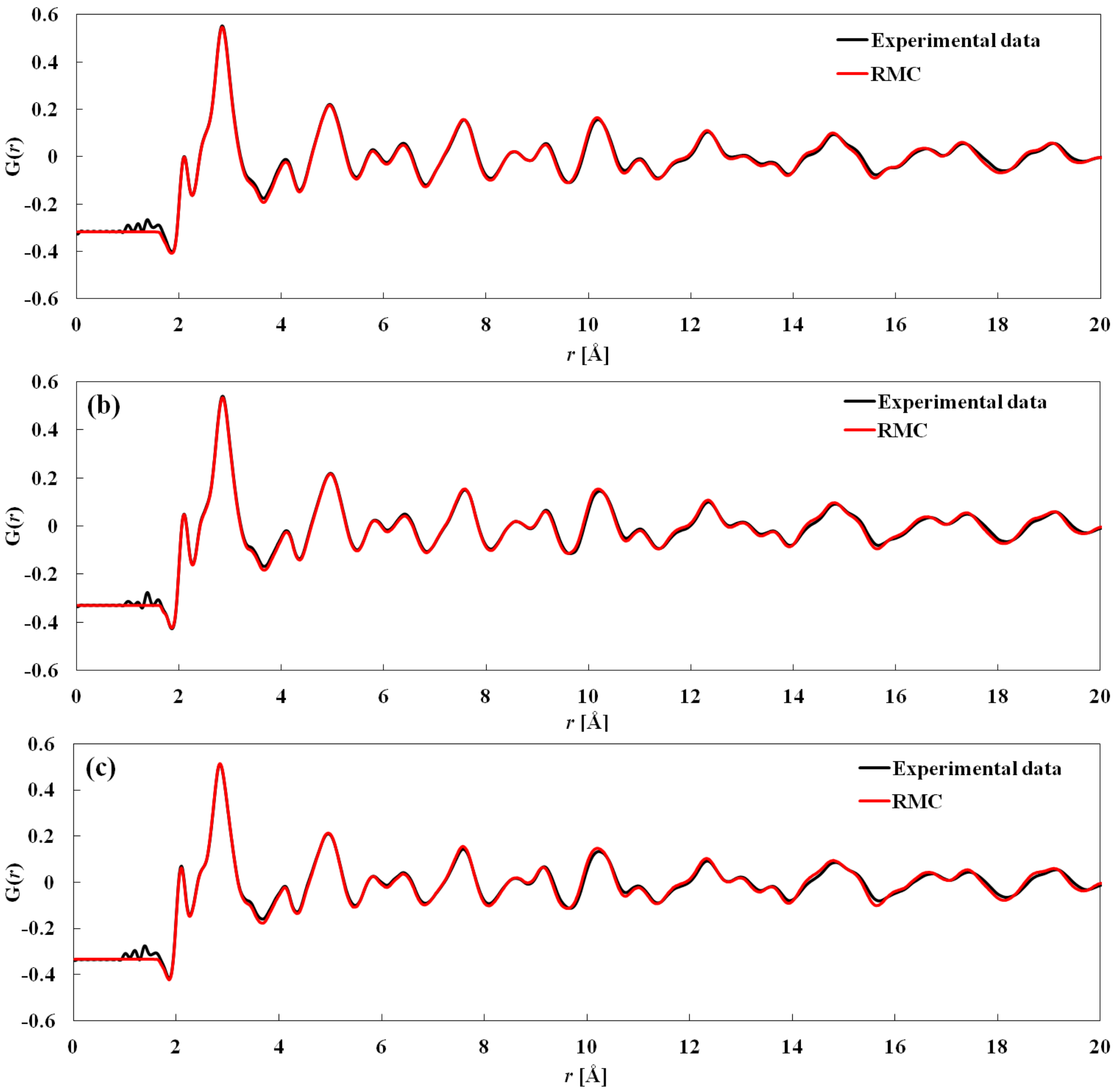

Fig. S6 RMC results for x = 0.55.
